# Effects of Simulated Video Consultation Training on Nursing Students’ Clinical Decision Making, Self-Confidence, and Anxiety: A Quasi-Experimental Study

**DOI:** 10.3390/nursrep16060181

**Published:** 2026-05-26

**Authors:** Glòria Tort-Nasarre, Alícia Baltasar-Bagué, Maria del Carmen Malagón-Aguilera, Mariona Vilar-Pont, Carla Camí, Glòria Reig-Garcia

**Affiliations:** 1 Department of Nursing and Physiotherapy, University of Lleida, 25198 Lleida, Spain; 2 Health Education, Nursing, Sustainability and Innovation Research Group (GREISI), Lleida Institute for Biomedical Research, Dr. Pifarré Foundation, IRB Lleida, 25198 Lleida, Spain; 3 Department of Nursing, University of Girona, 17003 Girona, Spain; 4 Catalan Institute of Health, 08007 Barcelona, Spain

**Keywords:** anxiety, clinical decision making, digital health education, nursing education, self-confidence, simulation-based education

## Abstract

**Background**: The expansion of telenursing in primary care requires undergraduate nursing programmes to prepare students for clinical decision making in virtual care. However, evidence on educational interventions integrating clinical reasoning with managing uncertainty, anxiety, and self-confidence in telenursing remains limited. **Objective**: This study aims to assess changes in nursing students’ self-confidence and anxiety related to clinical decision making following a simulation-based video consultation intervention, and to explore their satisfaction and perceived learning regarding this educational experience. **Methods**: A quasi-experimental pretest–post-test study with a comparison group was conducted at two public universities during the 2024–2025 academic year. Undergraduate nursing students (*N* = 115) were included. The intervention consisted of theoretical training on video consultations, complemented by structured simulated consultations based on primary care scenarios. The control group received no intervention. Outcomes were assessed before and after, with self-confidence and anxiety measured using the Nursing Anxiety and Self-Confidence with Clinical Decision Making (NASC-CDM^©^) scale. Satisfaction and perceived learning were evaluated in the intervention group using the Student Satisfaction and Self-Confidence in Learning Scale. **Results**: At baseline, students had high digital competence but limited preparedness for telenursing, with heterogeneous self-confidence and moderate anxiety in virtual decision making. After the intervention, the intervention group showed significant increases in self-confidence and reductions in anxiety, particularly in global clinical judgement and autonomous decision making. Students reported high satisfaction and perceived learning. **Conclusions**: Simulation-based video consultations may improve nursing students’ self-confidence and reduce anxiety in virtual care decision making, suggesting a potentially valuable strategy for integrating psychoeducational dimensions into nursing curricula on digital health.

## 1. Introduction

The rapid expansion of telenursing in primary care has reshaped nursing practice and highlighted the need for undergraduate nursing programmes to prepare students for virtual care delivery [1]. Consequently, undergraduate nursing programmes must incorporate educational strategies that prepare students for clinical decision making and patient interaction in virtual care environments [2].

Telenursing refers to nursing care provided via telecommunication and information technology products and services, with frequent contact through telephones or other communication methods [3]. Within this transformation, telenursing has become a core competence, requiring nurses to conduct remote assessments, follow-up and patient education, often in the absence of physical examinations [4,5]. Therefore, effective telenursing not only demands technological proficiency, but also advanced clinical reasoning, communication, and decision-making skills adapted for virtual contexts. These competencies should be systematically developed during undergraduate training to prepare students for contemporary practice [6,7].

However, current evidence suggests that many nursing programmes do not sufficiently address the complex competencies required for effective telenursing, leaving students with limited self-efficacy and preparation for remote practice [8,9]. A recent scoping review indicates that, despite the growing incorporation of digital health content into nursing curricula, important gaps remain in the scope, coherence, and evaluation of educational interventions, which may limit students’ preparedness for effective engagement in virtual care contexts [10]. While students generally value telehealth as an innovative care modality, they often report uncertainty and low confidence when managing virtual consultations independently [11,12]. This highlights the need for integrated educational approaches that enhance technological competence, foster positive attitudes, and strengthen confidence when conducting video consultations.

Although simulation is widely recognised as a pedagogical approach that supports the development of clinical and telehealth-specific skills by providing realistic and safe practice environments, recent scoping reviews highlight a clear lack of research on the use of simulated telehealth in nursing education [13]. Previous studies suggest that simulated teleconsultations are feasible and well received for developing communication and technical skills [14,15,16]. However, evidence remains limited regarding their impact on higher-order cognitive and emotional outcomes, particularly students’ confidence and anxiety related to clinical decision making during video consultations.

Clinical decision making in telenursing involves synthesising incomplete information, identifying priority problems and making timely judgements without direct physical assessment, which increase clinical uncertainty and cognitive demand. These complexities can heighten stress and anxiety, especially among students with limited clinical experience, potentially affecting performance [17,18]. Self-confidence and the ability to regulate anxiety are integral to safe decision making and have been identified as key psychoeducational variables influencing clinical performance in simulated settings. Recent evidence suggests that targeted telenursing training can enhance students’ self-confidence while reducing perceived barriers to virtual care [19].

Despite their importance, these factors have rarely been examined together in studies evaluating telenursing educational interventions. Specifically, simulation offers a controlled learning environment where students can practise clinical reasoning, communication and decision making while receiving feedback that supports confidence building and emotional regulation [8,16]. However, research to date has predominantly focused on perceived competence and satisfaction, with limited exploration of the joint effects on decision making, self-confidence and anxiety within simulated video consultation contexts. Given the increasing prevalence of virtual care and the centrality of clinical decision making to nursing practice, addressing this gap is essential for evidence-informed curriculum design.

This study responds to an internationally recognised educational gap in undergraduate nursing programmes by addressing the cognitive and emotional dimensions of clinical decision making in simulated video consultations. Although conducted in a single national context, the competencies examined and the pedagogical approach aligned with those required across healthcare systems undergoing similar digital transformation processes. As such, the findings may offer transferable evidence for the international nursing education literature.

Accordingly, this study aims to assess changes in nursing students’ self-confidence and anxiety related to clinical decision making following a simulation-based video consultation intervention, and to explore their satisfaction and perceived learning regarding this educational experience.

## 2. Materials and Methods

### 2.1. Study Design

A quasi-experimental pretest–post-test design with a comparison group was used in this study.

This manuscript was prepared in accordance with the Transparent Reporting of Evaluations with Non-randomised Designs (TREND) reporting guidelines [20]. A completed TREND checklist is provided in Appendix A.

### 2.2. Setting and Participants

The study was conducted at two public universities in Spain during the 2024–2025 academic year. Participants were undergraduate nursing students enrolled in the third years of either Family and Community Nursing at the University of Lleida (intervention group) or the Community Nursing Practicum at the University of Girona (comparison group). All students who met the inclusion criteria were invited by their own instructors to participate. The inclusion criteria were being enrolled in the corresponding course, attending all scheduled educational activities, and giving their informed consent. Students participating in academic mobility programmes or opting for single-assessment evaluation were excluded.

### 2.3. Educational Intervention

The intervention was designed as a structured educational programme aimed at developing competence in managing video consultations in primary care, with a specific focus on clinical decision making, self-confidence, and emotional regulation in telenursing contexts. It consisted of two sequential phases.

The first phase involved theoretical training delivered by a digital health expert, addressing the organisation of video consultations, communication skills in virtual care settings, and issues related to privacy and data confidentiality. This phase was complemented by a self-directed online learning module hosted on the institutional virtual learning platform, including scientific articles and practical guidelines related to video consultation practice. The total duration of this phase was 8 h.

The second phase consisted of structured simulated video consultations implemented in accordance with the Healthcare Simulation Standards of Best Practice™ [21], ensuring a psychologically safe learning environment throughout the briefing, simulation, and debriefing stages. Scenarios were based on a contextualised adaptation of the model proposed by Rutledge et al. [22], incorporating culturally relevant elements of the local healthcare system. Each student participated in five different simulation scenarios.

Each simulation lasted approximately 30 min and involved interaction with trained simulated patients through clinical interviews, remote assessment, decision making, and health education. Simulated patients were trained using standardised scripts, and scenarios were standardised in structure, learning objectives, and timing to ensure consistency and reproducibility.

Each session was followed by a structured debriefing lasting 20–30 min, focused on clinical reasoning, decision making, and emotional responses. Debriefings were conducted by nursing faculty trained in clinical simulation, following a predefined facilitation guide. The institutional virtual campus was used as the communication platform. A detailed description of the intervention is provided in Appendix B.

The control group did not receive the simulation-based intervention and served as a comparison to evaluate its impact. They continued with their usual academic activities and did not receive any additional educational input related to video consultations or telenursing during the study period.

### 2.4. Instruments and Data Collection

Data collection was conducted between January and September 2025. Participants in the intervention group completed questionnaires before and after the educational intervention, whereas the control group completed the questionnaires only once.

An ad hoc questionnaire was used to collect sociodemographic data and to assess individual indicators of digital competence and innovative capacity, including the understanding and use of telenursing systems, confidence in managing video consultations, the perceived ability to deliver care via teleassistance, and attitudes towards the use of digital technologies. These indicators were assessed at baseline and post-intervention in both the control and intervention groups.

Clinical decision making, self-confidence and anxiety were assessed using the Nursing Anxiety and Self-Confidence with Clinical Decision Making (NASC-CDM^©^) scale, in its Spanish version adapted by Medel et al. [23], with the original author’s permission. This instrument evaluates nursing students’ confidence and anxiety related to clinical decision making through two subscales (self-confidence and anxiety) and three dimensions: D1 (use of resources for information gathering and active listening), D2 (use of information to obtain a global perspective), and D3 (knowing and acting).

In the intervention group, satisfaction with the simulated video consultation methodology was assessed using the Student Satisfaction and Self-Confidence in Learning Scale (SCLS) in its validated Spanish version [24]. The SCLS is a 13-item instrument comprising two dimensions—satisfaction with current learning and self-confidence in learning—and has demonstrated good psychometric properties in nursing simulation contexts.

Internal consistency analyses were conducted for all NASC-CDM^©^ subscales and for the SCLS in the study sample using Cronbach’s alpha coefficients. The NASC-CDM^©^ subscales showed good to excellent internal consistency (α = 0.83–0.93). The SCLS also demonstrated adequate internal consistency overall. Item-level analysis identified that item 13 of the SCLS showed lower item-total correlation compared with the remaining items of the same subscale. Although Cronbach’s alpha slightly improved when this item was removed or reverse coded, the original validated scoring structure was retained to preserve comparability with previous validation studies.

Taken together, these instruments allowed the educational impact of telenursing simulation on nursing students to be analysed, with a specific focus on clinical decision making and the development of self-confidence.

### 2.5. Data Analysis

Data were analysed using IBM SPSS Statistics (version 29.0). Descriptive statistics summarised participants’ sociodemographic characteristics and baseline variables, including means and standard deviations for continuous variables and frequencies and percentages for categorical variables. The normality of continuous variables was assessed using the Shapiro–Wilk test, and parametric or non-parametric tests were applied accordingly.

Comparisons between centres (Lleida vs. Girona) for digital competence, self-confidence, and anxiety, as well as within-group pre–post changes in the intervention group, were performed using independent-samples *t*-tests, Mann–Whitney U tests, paired-samples *t*-tests, or Wilcoxon signed-rank tests, depending on the data distribution. Changes in digital competence and innovative capacity variables before and after the intervention were analysed using paired comparisons according to variable distribution.

Because the comparison group was assessed only once at baseline, no longitudinal or repeated-measures analyses could be performed across groups, nor could changes over time be directly compared between the intervention and control groups. This also prevented the use of multivariate approaches such as ANCOVA adjusting post-intervention outcomes for baseline values in both groups. Where possible, baseline differences between groups were partially accounted for through cross-sectional comparisons and adjusted analyses within the intervention group, although these approaches do not replace a controlled longitudinal design.

Student satisfaction with the simulation-based intervention was analysed descriptively using means and standard deviations for the two dimensions of the SCLS. All statistical tests were two-tailed, and a *p*-value < 0.05 was considered statistically significant.

### 2.6. Ethical Considerations

The study was approved by the University of Girona Research Ethics Committee (Code: CEBRU0060-24). Prior to the intervention, all participants received detailed information about the study objectives, methodology, potential benefits and risks, and were informed of their right to withdraw at any time without academic consequences. Written informed consent was obtained from all students.

Data were handled confidentially and anonymised. Each participant was assigned a unique identification code, and identifiable information was accessible only to the research team. Data were stored on the institutional cloud servers of the University of Lleida, in compliance with European data protection regulations. As a form of feedback and direct benefit, participants were offered a summary of the study results and access to published articles upon request.

## 3. Results

The results are presented in two main sections: baseline and post-intervention. Baseline results are organised under the following subsections: *Characteristics of the participants—Digital competence*, *Clinical decision making*, and *Differences by centre* (*Lleida* vs. *Girona*). Post-intervention results are reported in the subsections *Post-intervention results in the Lleida group* and *Student satisfaction with the simulation*.

### 3.1. Characteristics of the Participants—Digital Competence

A total of 115 students participated (53 from Girona, 62 from Lleida), 86.1% female, mean age 20 years (range 20–22). Only 2.6% had prior telemedicine training, and 30.7% were employed in healthcare; no significant differences were observed by the centre. Digital competence and innovative capacity were assessed through eight self-reported items addressing perceived understanding, use, and confidence in telenursing and information and communication technologies (ICT). At baseline, 33–28.7% of participants agreed or strongly agreed that they understood telenursing systems, and 59% reported confidence in using them. Confidence in managing telenursing consultations and providing effective care via teleassistance were more heterogeneous (27–29.6% agreement).

Regarding technology use, 37.4% were neutral about preferring new technologies, while 46.1% enjoyed trying them. Most participants reported high ease of technological learning (80% agreed or strongly agreed) and possessed digital skills for ICT-based learning (79.2% agreed or strongly agreed).

### 3.2. Clinical Decision Making

Nursing students’ anxiety and self-confidence in clinical decision making were assessed using the NASC-CDM^©^ scale, which includes 27 items on anxiety and 27 on confidence. Factor analysis confirmed a three-dimensional structure.

Regarding confidence, the highest-scoring items were: “I am confident and aware of my ability to recognize when to discuss or ask my clinical tutor for help in classifying client assessment results” (4.90 ± 1.02), “I am confident and aware of my ability to recognize when to discuss or ask my clinical tutor about interventions I am considering” (4.87 ± 1.06), and “I am confident and aware of my ability to use active listening skills when gathering information about a client’s current problem” (4.86 ± 1.20).

The lowest-scoring confidence item was: “I am confident and aware of my ability to identify the client’s full/global clinical picture rather than a part of it” (3.87 ± 1.06).

Regarding anxiety, the highest-scoring items were: “I am confident and anxious about my ability to make an autonomous clinical decision to solve a client’s problem” (3.37 ± 1.35), “I am confident and anxious about my ability to implement a specific intervention if the client has an urgent problem” (3.30 ± 1.27), and “I am confident and anxious about my ability to carry out an intervention based on instinct or intuition” (3.25 ± 1.30).

The lowest-scoring anxiety item was: “I am confident and anxious about my ability to incorporate the client’s personal aspects when making decisions in their best interest” (2.28 ± 1.14).

The 27 confidence and anxiety items were grouped according to the three dimensions proposed by the questionnaire (Table 1).

Table 2 presents item-level results for confidence and anxiety, as well as aggregated scores by dimension.

### 3.3. Differences by Centre (Lleida vs. Girona)

Students from Girona showed higher scores in confidence to manage telenursing consultations (55.6% vs. 25.7% agreed or strongly agreed; *p* = 0.010) and in the perceived ability to provide effective nursing care via teleassistance (45.3% vs. 32.4% agreed or strongly agreed; *p* = 0.025). In contrast, students from Lleida scored higher on “I enjoy trying new technologies” (80.6% vs. 56.5% agreed or strongly agreed; *p* = 0.040).

Regarding clinical decision-making outcomes, significant differences were observed in the confidence dimensions D2 and D3 and the anxiety dimensions D2 and D3. Girona students scored higher in confidence: D2 [30.4 (4.21) vs. 28.1 (5.09); *p* = 0.01] and D3 [30.1 (5.33) vs. 27.7 (5.35); *p* = 0.01]. In terms of anxiety, Lleida students scored higher: D2 [22.4 (6.06) vs. 19.9 (6.61); *p* = 0.03] and D3 [24.1 (6.22) vs. 20.9 (6.32); *p* < 0.001].

No significant differences were found in D1 between groups. Table 3 presents all items for each dimension according to the centre.

Between-group comparisons were limited to baseline data because the comparison group was not assessed post-intervention. Therefore, observed differences reflect initial group characteristics rather than intervention effects.

### 3.4. Post-Intervention Results in the Lleida Group

Regarding participants’ digital competence and innovative capacity, significant improvements were observed (*p* < 0.01) in the following variables: “Understanding of telenursing systems,” “Ability to use these systems,” “Confidence in managing telenursing consultations,” and “Perceived ability to provide effective nursing care via teleassistance”. In contrast, the variables “I prefer to use new technologies,” “I enjoy trying new technologies,” “I can easily learn new technologies,” and “I have digital skills for ICT-based learning” did not show significant changes after the intervention (Table 4).

After the intervention, confidence increased significantly (*p* < 0.01) across all dimensions: D1: from 60.7 (±10.3) to 65.5 (±7.54), mean change 4.85 (±10.2); D2: from 28.1 (±5.09) to 32.1 (±4.59), mean change 4.05 (±5.61); and D3: from 27.7 (±5.35) to 32.3 (±4.49), mean change 4.63 (±5.95).

**Figure 1:**
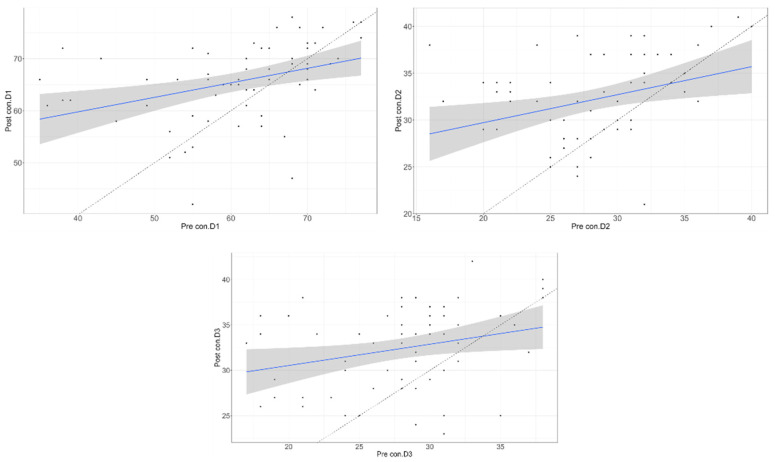
Changes in the three confidence dimensions after the intervention.

**Figure 2:**
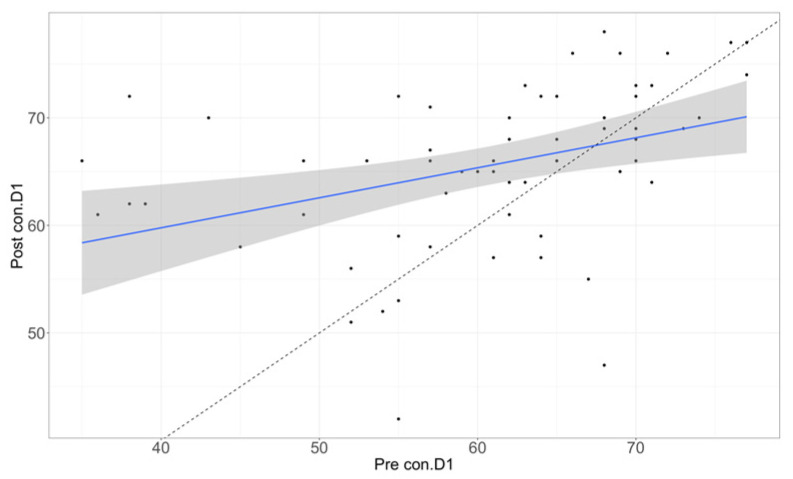

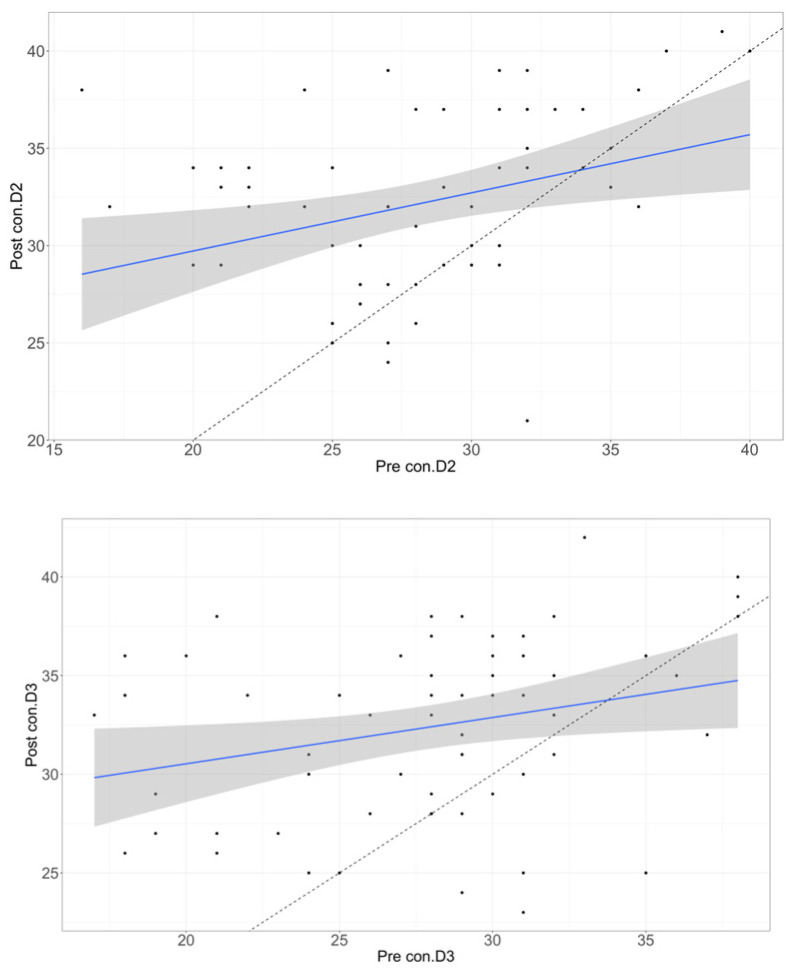
Changes in the three anxiety dimensions after the intervention.

Regarding anxiety, scores decreased significantly (*p* < 0.01) across all measured dimensions: D1: from 32.9 (±10.9) to 29.7 (±12.3), mean change −3.19 (±10.8); D2: from 22.4 (±6.06) to 19.0 (±5.97), mean change −3.39 (±5.85); and D3: from 24.1 (±6.22) to 19.9 (±6.27), mean change −4.24 (±6.52). Thus, the intervention demonstrated a consistently positive impact across all confidence dimensions, with greater improvements observed in students who initially had lower scores. Similarly, the intervention’s positive effect on anxiety reduction was evident across all three dimensions, with the largest benefit seen in participants with higher baseline anxiety scores (Figure 1 and Figure 2).

### 3.5. Student Satisfaction with the Simulation

Students’ satisfaction with the simulation was measured using the SCLS, which includes 13 items grouped into two dimensions: D1—Satisfaction with overall learning and D2—Self-confidence in learning. The minimum and maximum scores are 5 and 25 for D1, and 8 and 40 for D2, respectively.

**Figure 3:**
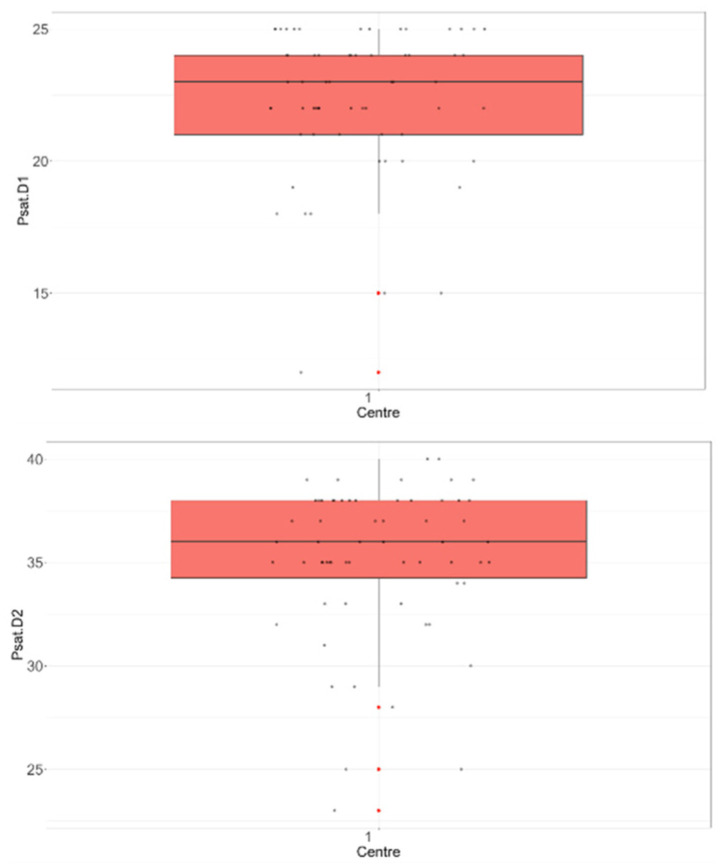
Students’ satisfaction with the simulation-based intervention.

Satisfaction with the simulation-based intervention showed high scores in both dimensions, with a mean of 22.3 (±2.75) for D1 and 35.2 (±3.67) for D2 (Figure 3).

## 4. Discussion

The findings of this study suggest that nursing students enter their training with a generally high level of digital competence and a positive attitude towards the use of information and communication technologies, yet with limited preparation for telenursing practice. Despite the perceived ease of learning new technologies and confidence in ICT-based learning, prior to the intervention only a small proportion of participants reported an adequate understanding of telenursing systems or felt confident in managing video consultations and delivering effective care through teleassistance. This gap between general digital literacy and specific clinical competence has been widely described in the literature, which highlights that technological familiarity is a necessary but insufficient condition for ensuring safe clinical practice in virtual care settings [22,25]. In this regard, the present findings provide additional evidence that extends existing literature, suggesting that the lack of structured training in telenursing remains a relevant limitation in undergraduate nursing programmes, despite the growing integration of virtual care into clinical practice. This interpretation is consistent with previous studies emphasising the need for targeted digital health education in nursing curricula [10].

Analysis of baseline clinical decision-making patterns suggested high levels of confidence in skills related to information gathering, active listening, and recognising the need to seek support, alongside lower confidence in processes requiring information integration to construct a comprehensive clinical picture and make autonomous decisions. These dimensions are generally considered central to clinical reasoning according to Tanner’s Clinical Judgment Model [26] and the clinical reasoning frameworks proposed by Levett-Jones et al. [27], which identify interpretation and information integration as the most complex—and often most fragile—processes among students with limited clinical experience. In telenursing contexts, these challenges may be further amplified by the absence of direct physical examination and the reliance on mediated information, which can complicate the interpretation and synthesis phases of clinical judgement, in telehealth studies highlighting challenges in clinical assessment, care quality, and workflow in remote consultations [28].

The findings also highlight a complex relationship among self-confidence, anxiety, and autonomy in clinical decision making. Prior to the intervention, the coexistence of moderate to high confidence with significant anxiety in situations requiring autonomous decision making—particularly in urgent contexts—suggests that anxiety does not necessarily reflect a lack of competence, but rather an awareness of clinical risk and professional responsibility. This interpretation is consistent with recent evidence indicating that confidence and anxiety operate as interrelated—and not opposing—components of clinical judgement in high-uncertainty contexts [29]. This perspective may allow anxiety to be reconceptualised not as a competence deficit, but as an expected response in the early stages of professional development, where awareness of clinical risk precedes the consolidation of safe action patterns [30]. Similarly, research on nursing decision making has shown that uncertainty and anxiety are inherent components of the decision-making process, particularly in complex contexts characterised by limited information [31].

The differences observed between participating centres indicates the role of the educational context as a potential moderator of clinical reasoning and emotional regulation. Prior to the intervention, students from Girona demonstrated higher confidence in dimensions related to information integration and autonomous decision making, whereas students from Lleida reported higher anxiety in these same dimensions, despite expressing a more favourable attitude towards the use of new technologies. This pattern suggests that technological predisposition alone does not guarantee greater clinical security, and that factors such as prior exposure to clinical situations, pedagogical approaches, and the educational climate may have a relevant influence on the development of clinical judgement.

Following the simulation-based video consultation intervention, the findings suggest a pattern of improvement in perceived competence in telenursing and clinical decision making. In line with this, changes were observed in understanding and use of telenursing systems, as well as in confidence when managing teleassistance consultations, while general attitudes towards technology remained stable.

This pattern may suggest that the intervention was more closely associated with clinical and professional competencies related to telenursing practice than with broader technological dispositions, supporting the consideration of simulation as a potentially targeted educational strategy rather than a generic digital training tool, consistent with previous evidence on the role of simulation in developing clinical and non-technical skills in digital and telehealth contexts [32,33].

Regarding clinical decision making, the intervention was associated with a significant increase in self-confidence and a consistent reduction in anxiety across all dimensions of clinical reasoning, with particularly pronounced effects among students with lower baseline scores. These findings suggest that simulated video consultations may function as a cognitively and emotionally safe learning environment, enabling students to practise decision making in conditions of uncertainty without the risks inherent to real clinical practice. This progressive exposure to complex situations in a safe learning environment may represent a defining strength of simulation-based pedagogy, particularly in telehealth contexts [32,34].

Finally, the reduction in initial differences between centres observed after the intervention in the experimental group suggests that simulation-based educational strategies may have the potential to mitigate educational inequalities and may contribute to more homogeneous development of students’ clinical and emotional competencies, although these findings should be interpreted with caution given the quasi-experimental design and baseline differences between centres. From an educational perspective, these findings support incorporating systematic and intentional simulation-based interventions into nursing curricula as a potentially valuable pedagogical strategy to support safe, autonomous, and emotionally regulated clinical decision making in telenursing contexts. This is in line with current recommendations for the integration of digital health and simulation in nursing education [35]. In this respect, the progressive integration of such interventions could contribute to more equitable training aligned with the demands of professional practice and with standards of quality and safety in healthcare.

### Limitations and Strengths

This study has limitations that should be considered. The absence of randomisation may introduce selection bias, as group allocation was determined by institutional affiliation rather than by chance. However, the students in the two groups were drawn from separate institutions, meaning that they did not know each other and did not share classes, which substantially reduced the risk of contamination between groups.

The comparison group was assessed only at baseline, with no follow-up measurements collected. This prevented the use of longitudinal or repeated-measures analyses across groups and limited the possibility of applying multivariate approaches to adjust post-intervention outcomes for baseline differences. Consequently, changes observed in the intervention group cannot be fully disentangled from potential external influences, and causal interpretations should be made with caution. This design constraint also limited the depth of between-group comparisons, which could only be conducted cross-sectionally at baseline.

In addition, item 13 of the SCLS showed lower internal consistency compared with the remaining items of the same dimension. Although the original validated scoring structure was maintained, this finding should be considered in future research.

Assessment of long-term knowledge retention, which typically requires a longitudinal component, was not feasible due to logistical constraints.

Nevertheless, the study addresses an underexplored area in nursing education by examining clinical decision making alongside self-confidence and anxiety in simulated video consultations. The use of validated instruments enabled an integrated assessment of cognitive and emotional dimensions, while the inclusion of two institutions and a clearly described, theory-informed simulation intervention enhances transparency, reproducibility, and relevance for contemporary digital healthcare curricula.

## 5. Conclusions

This study suggests that a structured simulation-based video consultation intervention may be associated with increased self-confidence and reduced anxiety related to clinical decision making among nursing students. However, these findings should be interpreted with caution given the quasi-experimental design and the inherent limitations they entail, including the absence of randomisation and the potential influence of confounding variables.

The results indicate that targeted educational strategies may support both cognitive and emotional processes involved in decision making under conditions of clinical uncertainty in telenursing contexts, although further research using more robust designs is needed to confirm these effects. In addition, students reported high levels of satisfaction and perceived learning, suggesting good acceptability of the intervention.

Overall, simulated video consultation appears to be a pedagogically relevant approach for supporting the development of clinical reasoning in virtual care settings. Nevertheless, the extent to which these findings can be generalised beyond the study context remains limited, and future studies should aim to replicate these results across different educational settings and with larger, more diverse samples.

## Figures and Tables

**Table 1:** Confidence and anxiety items grouped according to the three dimensions.

Dimension	Confidence Mean (SD)	Anxiety Mean (SD)
D1—Use of resources for information gathering and active listening	61.2 (9.70)	31.9 (11.1)
D2—Use of information to obtain a global perspective	29.1 (4.86)	21.2 (6.42)
D3—Knowledge and action	28.8 (5.46)	22.7 (6.44)

**Table 2:** Confidence and anxiety results.

**D1—** **Use of resources for information gathering and active listening (13 items)**			
**Item**	**Summary Statement**	**Confidence** **Mean (SD)**	**Anxiety** **Mean (SD)**
1	Detect important client information patterns.	4.24 (0.86)	3.03 (1.13)
2	Identify relevant clinical information.	4.29 (0.88)	2.86 (1.11)
4	Recall prior knowledge related to the problem.	4.30 (0.93)	2.79 (1.17)
6	Interpret a specific finding from the assessment.	4.04 (0.99)	3.10 (1.22)
8	Recognise the need to ask for help regarding assessment results.	4.90 (1.02)	2.45 (1.20)
9	Use active listening skills.	4.86 (1.20)	2.29 (1.14)
10	Evaluate nonverbal communication.	4.63 (1.11)	2.40 (1.23)
11	Recognise the need to review protocols or literature.	4.63 (1.11)	2.51 (1.20)
12	Assess information provided by the family or partner.	4.83 (0.99)	2.44 (1.18)
13	Apply knowledge of anatomy and physiology.	4.08 (1.09)	3.21 (1.29)
16	Recognise relevant information during shift handover.	4.62 (1.05)	2.68 (1.20)
18	Formulate additional questions for the client.	4.66 (1.02)	2.57 (1.14)
19	Correlate physical findings and non-verbal cues.	4.42 (0.99)	2.71 (0.98)
**D2—Use of information to obtain a global perspective (7 items)**			
**Item**	**Summary statement**	**Confidence** **Mean (SD)**	**Anxiety** **Mean (SD)**
3	Identify the client’s complete clinical picture.	3.87 (1.06)	3.23 (1.25)
5	Decide the most appropriate priority alternative.	4.05 (0.95)	3.16 (1.30)
15	Analyse the risks of interventions.	4.37 (1.05)	3.18 (1.10)
17	Make an autonomous clinical decision.	4.10 (1.17)	3.37 (1.35)
21	Interpret diagnostic tests to inform decisions.	4.06 (1.16)	3.21 (1.20)
23	Keep an open mind to different explanations.	4.64 (1.15)	2.38 (1.14)
27	Consider an intervention because it “seems” correct.	3.91 (1.20)	3.20 (1.30)
**D3—Knowledge and action (7 items)**			
**Item**	**Summary statement**	**Confidence** **Mean (SD)**	**Anxiety** **Mean (SD)**
7	Evaluate whether the clinical decision improves outcomes.	4.31 (1.10)	3.02 (1.26)
14	Perform an intervention based on intuition.	4.16 (0.99)	3.25 (1.30)
20	Implement an intervention in response to an urgent problem.	4.17 (1.10)	3.30 (1.27)
22	Recognise the need to ask for help regarding interventions.	4.87 (1.06)	2.32 (1.17)
24	Ask the client’s family or partner.	4.87 (1.01)	2.36 (1.16)
25	Assess the impact of the decision on client satisfaction.	4.62 (1.12)	2.45 (1.19)
26	Incorporate the client’s personal aspects into the decision.	4.68 (1.02)	2.28 (1.14)

**Table 3:** Pre-intervention results by group: self-confidence and anxiety in clinical decision making.

Variable	Total (*N* = 115) Mean (SD)	Lleida (*n* = 62) Mean (SD)	Girona (*n* = 53) Mean (SD)	*p* Global
**Self-confidence (items)**				
Confidence 1	4.24 (0.86)	4.16 (0.93)	4.34 (0.78)	0.266
Confidence 2	4.29 (0.88)	4.15 (0.94)	4.45 (0.77)	0.056
Confidence 3	3.87 (1.06)	3.74 (1.09)	4.02 (1.01)	0.159
Confidence 4	4.30 (0.93)	4.13 (0.93)	4.51 (0.89)	0.027 *
Confidence 5	4.05 (0.95)	3.90 (0.94)	4.23 (0.95)	0.070
Confidence 6	4.04 (0.99)	3.87 (1.03)	4.25 (0.90)	0.040 *
Confidence 7	4.31 (1.10)	4.18 (1.24)	4.47 (0.91)	0.145
Confidence 8	4.90 (1.02)	4.95 (1.05)	4.85 (0.99)	0.590
Confidence 9	4.86 (1.20)	4.81 (1.20)	4.92 (1.21)	0.601
Confidence 10	4.63 (1.11)	4.52 (1.11)	4.77 (1.10)	0.217
Confidence 11	4.63 (1.11)	4.55 (1.24)	4.72 (0.95)	0.410
Confidence 12	4.83 (0.99)	4.87 (1.11)	4.77 (0.85)	0.595
Confidence 13	4.08 (1.09)	3.87 (1.12)	4.32 (1.01)	0.026 *
Confidence 14	4.16 (0.99)	4.03 (1.02)	4.30 (0.93)	0.142
Confidence 15	4.37 (1.05)	4.21 (1.07)	4.55 (0.99)	0.083
Confidence 16	4.62 (1.05)	4.63 (1.09)	4.60 (1.01)	0.898
Confidence 17	4.10 (1.17)	3.87 (1.23)	4.38 (1.04)	0.019 *
Confidence 18	4.66 (1.02)	4.47 (1.08)	4.89 (0.89)	0.025 *
Confidence 19	4.42 (0.99)	4.24 (1.05)	4.62 (0.88)	0.037 *
Confidence 20	4.17 (1.10)	4.13 (1.08)	4.21 (1.13)	0.706
Confidence 21	4.06 (1.16)	3.79 (1.06)	4.38 (1.20)	0.007 *
Confidence 22	4.87 (1.06)	4.94 (1.17)	4.79 (0.91)	0.463
Confidence 23	4.64 (1.15)	4.53 (1.13)	4.77 (1.17)	0.265
Confidence 24	4.87 (1.01)	4.92 (1.03)	4.81 (1.00)	0.570
Confidence 25	4.62 (1.12)	4.56 (1.14)	4.68 (1.11)	0.586
Confidence 26	4.68 (1.02)	4.71 (1.11)	4.64 (0.92)	0.719
Confidence 27	3.91 (1.20)	3.77 (1.30)	4.08 (1.05)	0.172
**Anxiety (items)**				
Anxiety 1	3.03 (1.13)	3.19 (1.13)	2.83 (1.10)	0.084
Anxiety 2	2.86 (1.11)	2.94 (1.16)	2.77 (1.05)	0.433
Anxiety 3	3.23 (1.25)	3.37 (1.26)	3.08 (1.24)	0.208
Anxiety 4	2.79 (1.17)	3.02 (1.12)	2.53 (1.17)	0.025 *
Anxiety 5	3.16 (1.30)	3.35 (1.27)	2.92 (1.31)	0.078
Anxiety 6	3.10 (1.22)	3.24 (1.11)	2.92 (1.33)	0.172
Anxiety 7	3.02 (1.26)	3.18 (1.29)	2.83 (1.20)	0.138
Anxiety 8	2.45 (1.20)	2.48 (1.21)	2.42 (1.20)	0.761
Anxiety 9	2.29 (1.14)	2.39 (1.22)	2.17 (1.03)	0.303
Anxiety 10	2.40 (1.23)	2.55 (1.26)	2.23 (1.19)	0.162
Anxiety 11	2.51 (1.20)	2.50 (1.21)	2.53 (1.20)	0.900
Anxiety 12	2.44 (1.18)	2.37 (1.22)	2.53 (1.14)	0.476
Anxiety 13	3.21 (1.29)	3.45 (1.34)	2.92 (1.17)	0.026 *
Anxiety 14	3.25 (1.30)	3.50 (1.26)	2.96 (1.30)	0.027 *
Anxiety 15	3.18 (1.10)	3.40 (1.02)	2.92 (1.14)	0.020 *
Anxiety 16	2.68 (1.20)	2.89 (1.17)	2.43 (1.18)	0.043 *
Anxiety 17	3.37 (1.35)	3.55 (1.39)	3.15 (1.28)	0.113
Anxiety 18	2.57 (1.14)	2.73 (1.26)	2.40 (0.97)	0.115
Anxiety 19	2.71 (0.98)	2.81(0.97)	2.60 (0.99)	0.272
Anxiety 20	3.30 (1.27)	3.52 (1.34)	3.04 (1.14)	0.041 *
Anxiety 21	3.21 (1.20)	3.44 (1.24)	2.94 (1.12)	0.027 *
Anxiety 22	2.32 (1.17)	2.42 (1.19)	2.21 (1.13)	0.332
Anxiety 23	2.38 (1.14)	2.47 (1.20)	2.28 (1.06)	0.383
Anxiety 24	2.36 (1.16)	2.42 (1.19)	2.28 (1.12)	0.529
Anxiety 25	2.45 (1.19)	2.53 (1.21)	2.36 (1.18)	0.438
Anxiety 26	2.28 (1.14)	2.37 (1.15)	2.17 (1.14)	0.349
Anxiety 27	3.20 (1.30)	3.37 (1.37)	3.00 (1.19)	0.123
**Anxiety dimensions**				
D1	31.9 (11.1)	32.9 (10.9)	30.6 (11.3)	0.268
D2	21.2 (6.42)	22.4 (6.06)	19.9 (6.61)	0.038 *
D3	22.7 (6.44)	24.1 (6.22)	20.9 (6.32)	0.008 *
**Self-confidence dimensions**				
D1	61.2 (9.70)	60.7 (10.3)	61.8 (8.97)	0.522
D2	29.1 (4.86)	28.1 (5.09)	30.4 (4.31)	0.011 *
D3	28.8 (5.46)	27.7 (5.35)	30.1 (5.33)	0.018 *

*p*-values marked with * indicate statistical significance (*p* < 0.05).

**Table 4:** Changes in digital competence and innovative capacity responses pre- and post-intervention.

Variable	Other_Pre → Other_Post	Other_Pre → Agree_Post	Other_Pre → Other_Post	Agree_Pre → Agree_Post	*p*-Value
I can understand telenursing systems.	2	22	2	36	0.0001
I can use telenursing systems.	2	26	0	34	<0.0001
I am confident in managing telenursing consultations effectively.	7	39	0	16	<0.0001
I feel capable of providing effective nursing care via teleassistance.	8	34	1	19	<0.0001
I prefer to use new technologies.	26	9	12	15	0.6625
I enjoy trying new technologies.	9	3	5	45	0.7237
I can easily learn new technologies.	4	9	2	47	0.0704
I have digital skills for ICT-based learning.	6	10	3	43	0.0961

Agree includes both ‘Agree’ and ‘Strongly Agree’ responses.

**Figure A1:**
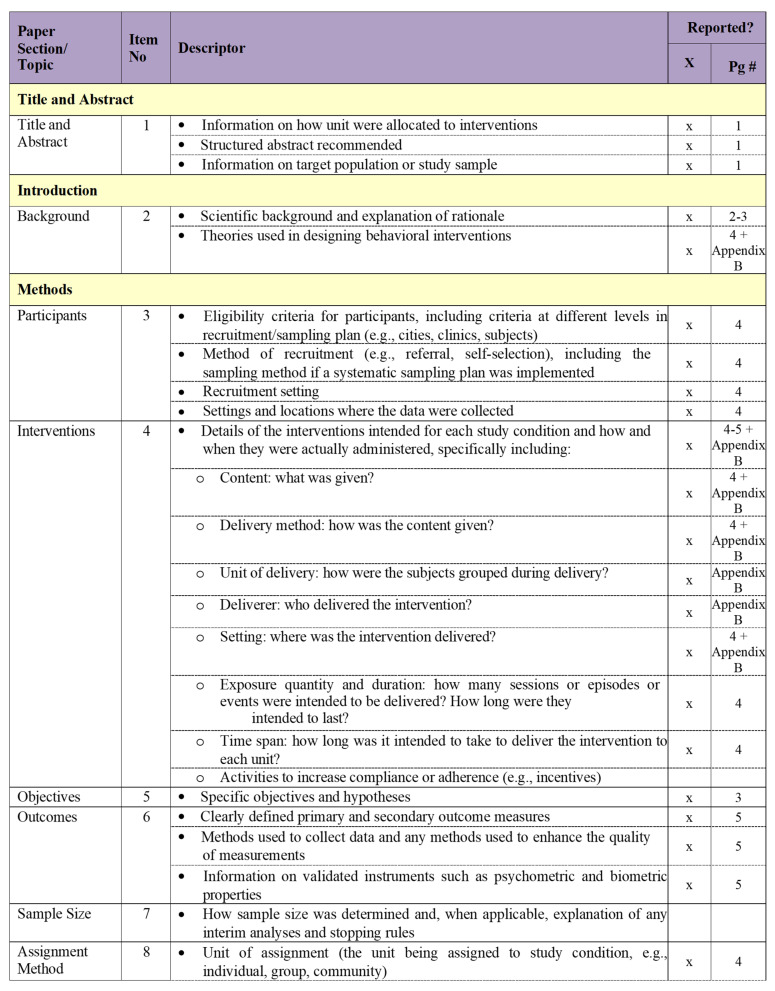

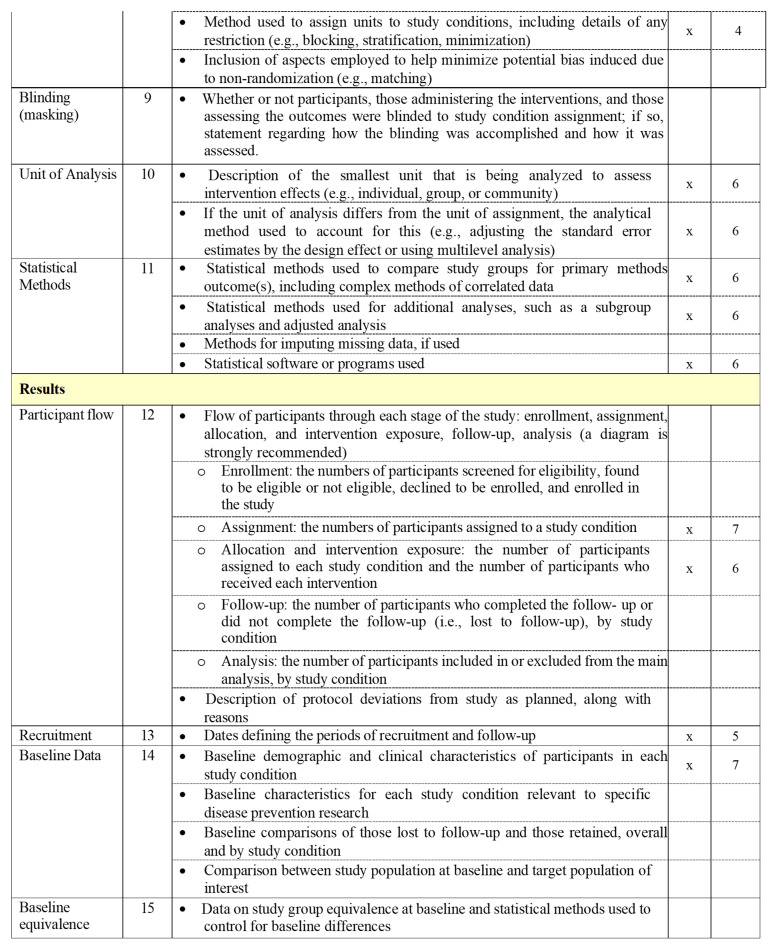

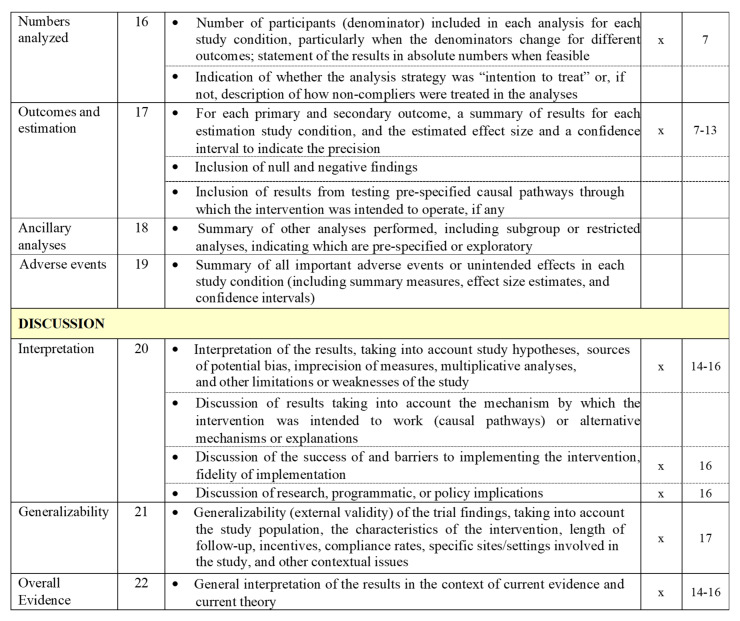
TREND checklist [20].

## Data Availability

The data presented in this study are openly available in the CORA Repositori de Dades de Recerca at https://doi.org/10.34810/data2939.

## Public Involvement Statement

No public involvement in any aspect of this research.

## Guidelines and Standards Statement

This manuscript was drafted against the Transparent Reporting of Evaluations with Non-randomised Designs (TREND) statement for reporting non-randomised intervention studies.

## Abbreviations

The following abbreviations are used in this manuscript:

ICT	Information and communication technologies
NASC-CDM^©^	Nursing Anxiety and Self-Confidence with Clinical Decision Making
SCLS	Student Satisfaction and Self-Confidence in Learning Scale
TREND	Transparent Reporting of Evaluations with Non-randomised Designs

## Appendix B. Simulation-Based Video Consultation Intervention

**Theoretical framework**

The intervention was informed by the Four P’s of Telehealth framework [22] and adapted to the Catalan healthcare and academic context. The model emphasises preparation, patient-centred communication, clinical decision making, and professional reflection in telehealth encounters.

**Educational components**

All participants received identical theoretical input on digital health and telenursing fundamentals, including organisation of video consultations, communication skills, privacy and confidentiality, and ethical considerations. Self-directed learning materials (scientific articles and practical guidelines) were provided through the virtual learning platform.

**Simulation structure**

The simulation was delivered to the intervention group using standardised video consultation scenarios situated in primary care. Each simulation followed three structured phases:

1. Briefing

Introduction to learning objectives, clarification of roles, orientation to the virtual consultation environment, and review of clinical and communication expectations.

2. Simulation

Individual or paired participation in a live video consultation with a trained simulated patient, conducted in a virtual primary care setting. Students managed the consultation autonomously, assuming a professional nursing role.

The simulation scenarios were based on standardised clinical cases commonly encountered in primary care telehealth, designed to elicit clinical reasoning, communication skills, and emotional regulation under conditions of uncertainty. In addition, scenarios deliberately incorporated elements related to the acceptability and perceived appropriateness of video consultations from the user’s perspective, including preferences for virtual versus face-to-face care, perceived limitations of remote assessment (e.g., need for physical examination), and concerns regarding privacy, confidentiality, and technological constraints.

Cases included: (a) follow-up of chronic conditions (e.g., chronic obstructive pulmonary disease) focusing on symptom assessment, medication management, and psychosocial factors; (b) management of sensitive or stigmatised health issues (e.g., urinary incontinence) requiring empathic communication and patient education; (c) post-intervention follow-up (e.g., post-bariatric surgery) involving assessment of warning signs, health education, visual inspection through the screen, and decisions regarding the need for face-to-face evaluation; and (d) health behaviour change counselling (e.g., smoking cessation) including discussion of user preferences for virtual care.

During the simulation, students conducted a structured remote assessment, prioritised nursing problems, made clinical decisions without direct physical examination, provided tailored health education, and negotiated follow-up plans. The scenarios integrated both cognitive demands (clinical judgement, prioritisation, decision making) and emotional demands (management of anxiety, uncertainty, and patient vulnerability) inherent to video-based care.

3. Debriefing

A structured, facilitated group debriefing was conducted immediately after each simulation by faculty trained in simulation methodology. The debriefing was explicitly aligned with the study outcomes and focused on reflective learning related to clinical decision making, self-confidence, and anxiety.

Debriefing discussions guided students to reflect on their clinical reasoning and prioritisation processes, decision making under uncertainty, and use of available information in the absence of physical examination. Emotional responses experienced during the simulation, including stress, uncertainty, and confidence fluctuations, were explicitly explored to promote emotional awareness and regulation. Communication strategies, patient engagement, perceived acceptability of the video consultation, and management of privacy and ethical issues were also discussed.

Facilitators used open-ended questions and reflective prompts to encourage self-assessment, peer feedback, and identification of learning needs. This process aimed to reinforce confidence, normalise anxiety associated with telehealth decision making, and consolidate learning in a psychologically safe environment.

**Simulation scenarios**

Standardised cases reflected common primary care situations managed through telehealth, including chronic disease management, psychosocial vulnerability, post-intervention monitoring, and health behaviour change. All scenarios were designed to ensure consistency across sessions while allowing individual clinical judgement and explicit reflection on the appropriateness and limits of video consultation.

**Facilitators and fidelity**

Simulations were facilitated by experienced nursing faculty trained in clinical simulation methodology. Standardised patients followed detailed role scripts to ensure consistency. The intervention was implemented using the same structure, timing, learning objectives, and assessment criteria across participating institutions.

**Educational aims**

The intervention aimed to support the development of clinical decision making, self-confidence, and emotional regulation in telehealth contexts, while providing a safe environment for practice, feedback, and reflective learning.
